# COVID-19 farm outbreaks in Ontario, January–December 2020

**DOI:** 10.14745/ccdr.v49i05a06

**Published:** 2023-05-01

**Authors:** Hetal Patel, Ana Ulloa, Sarah Buchan, Mariana Abdulnoor, Jonathan Gubbay, Michelle Murti

**Affiliations:** 1 Dalla Lana School of Public Health, University of Toronto, Toronto, ON; 2 Public Health Ontario, Toronto, ON; 3 Department of Laboratory Medicine and Pathology, Temerty Faculty of Medicine, University of Toronto, Toronto, ON

**Keywords:** COVID-19, farm, workers, outbreaks, workplace

## Abstract

**Background:**

Farm workers are critical to Ontario’s food supply chain as they grow and harvest the food that Ontario relies on; however, they are subject to several occupation-related coronavirus disease 2019 (COVID-19) transmission risk factors. We describe the epidemiology of farm outbreaks in Ontario over the first calendar year of the pandemic and explore trends in outbreaks by season and type of farm.

**Methods:**

Data pertaining to farm outbreaks in Ontario from January 1 to December 31, 2020, and their associated laboratory-confirmed cases were extracted from the provincial database. Outbreaks were characterized by size, season, farm type and duration. Cases were characterized by age, gender, medical risk factors, clinical presentation and outcomes.

**Results:**

There were 64 farm outbreaks associated with 2,202 confirmed cases of COVID-19 in Ontario during 2020. The majority of outbreaks occurred in spring (n=25, 39.1%) and fall (n=25, 39.1%). The fewest outbreaks occurred in the summer (n=6, 9.4%), corresponding with low community rates during that time, and the majority of these were in greenhouse farms (n=5, 83.3%). The median outbreak size was 14.5 cases (range: 1–240), and the median duration was 23 days (range: 0–128). Among cases, most were male (83.2%), the median age was 35 years, 10.0% had one or more comorbidities, 31.2% were asymptomatic, 16 required hospitalization and three died.

**Conclusion:**

Farm outbreaks were a source of COVID-19 transmission and illness in 2020, particularly in the spring and fall. Outbreaks continued in greenhouse farms despite lower summer community transmission.

## Introduction

Severe acute respiratory syndrome coronavirus 2 (SARS-CoV-2) can be spread when infectious respiratory particles are inhaled by individuals or deposited on their mucosal surfaces ((1)). The risk of transmission is higher as the source-to-receptor distance decreases, which is common when working and/or living with others that are infected with coronavirus disease 2019 (COVID-19) ((1)). During the COVID-19 pandemic, agricultural workers were deemed an essential workforce and farms remained open and in-person due to their necessary role in growing and harvesting food ((2)). Farm workers faced unique challenges, increasing their risk of COVID-19 infection compared to other essential workers. Farm work is often in close proximity without physical barriers. This includes work in indoor greenhouses, which account for 32% of farms in Ontario ((3)). Greenhouse farms differ in humidity, temperature and ventilation compared with outdoor fields, and these conditions can make greenhouses a favourable environment for viral transmission ((4)). In addition, temporary foreign farm workers, which make up 31% of employees on farms in Ontario, may also share transportation and living quarters, and face language barriers, lower income and decreased access to healthcare services, which make them more susceptible to occupational risks such as COVID-19 ((3,5,6)).

In response to the unique concerns faced by farm workers during the COVID-19 pandemic, the Ontario Ministry of Agriculture, Food and Rural Affairs first introduced the “Enhanced Agri-Food Workplace Protection Program” in May 2020 to help farms improve the health and safety of agri-food workers in Ontario during the COVID-19 pandemic ((7)). The Ontario Ministry of Health first developed the “COVID-19 Guidance: On-Farm Outbreak Management” in September 2020 that provides recommendations for safe practices on worksites, transportation and shared accommodations ((8)). Vaccine distribution for COVID-19 in Ontario began in December 2020; but at the time, vaccines were only eligible for certain populations. Farm workers were not eligible for the vaccine until phase 3 of the vaccine rollout in Ontario, around August 2021 ((9)).

The objective of this analysis was to describe the epidemiology of COVID-19 outbreaks in farms in Ontario in the pre-vaccine year of the pandemic, for outbreaks with a start date between January 1 and December 31, 2020, all cases associated with these outbreaks up to January 31, 2021, and trends in outbreaks by season and type of farm (i.e. indoor greenhouse vs. outdoor field).

## Methods

### Data source

We obtained data on COVID-19 outbreaks on farms and laboratory-confirmed COVID-19 cases linked to those outbreaks from the Public Health Case and Contact Management Solution (CCM); a dynamic disease reporting system for COVID-19 case and contact management in Ontario. We also obtained data on laboratory-confirmed COVID-19 cases in the general Ontario population. Data were entered by staff at the 34 local public health units (PHU) and digitally extracted by Public Health Ontario in February 9, 2021.

### Outbreak definitions and analysis

Prior to the development of a provincial definition for farm outbreaks, outbreak declaration was at the determination of the local PHU investigating cases associated with a farm. As of September 2020, the Ontario Ministry of Health issued guidance defining a COVID-19 on-farm outbreak. A COVID-19 on farm outbreak is defined as “one case (of COVID-19) in a congregate living area or two cases of COVID-19 (in the workplace), either asymptomatic or symptomatic, and where there is evidence of COVID-19 transmission in either the congregate living area or the workplace” ((8)). Outbreaks with no outbreak-associated confirmed cases were removed from the analysis (n=2). Outbreaks were included in the study if their start date was between January 1, 2020, and December 31, 2020.

Outbreak start date was determined by the episode date of the first outbreak case; if this date was unknown or missing, the outbreak reported date was used, followed by the outbreak created date. Episode date for cases is based on an estimate of the best date of disease onset and is calculated using a hierarchy based on the date of symptom onset, specimen collection/test date or the date reported to the PHU.

Outbreaks were characterized by PHU, size (i.e. number of confirmed cases linked to the outbreak by the PHU) and duration (i.e. the time from the episode date of the first case to the episode date of the last case linked to the outbreak, up to January 2021). A manual review of farm outbreak locations was conducted to classify farms with greenhouses, given their additional risk for COVID-19 as crowded, indoor environments.

Outbreaks were also further categorized by season based on the outbreak start date. Spring outbreaks were those starting between March 20 and June 19, 2020; summer outbreaks those starting between June 20 and September 21, 2020; and fall outbreaks those starting between September 22 and December 20, 2020. Winter was removed from the analysis as there were limited data for this season in 2020 ((10)).

### Outbreak-associated cases

Laboratory-confirmed COVID-19 cases linked to farm outbreaks were included if their episode date was between January 1, 2020, and January 31, 2021, to include cases associated with outbreaks that were still open after December 31, 2020. Outbreaks were considered closed if they had a “declared over date” in CCM or if it had been five months since the outbreak start date. As of data extraction time, five included outbreaks remained open. Cases were characterized by age, gender, medical risk factors (including presence of one or more comorbidities and high-risk status), symptoms, outcomes and PHU where the outbreak occurred. Comorbidities included anemia, asthma, chronic obstructive pulmonary disease (COPD), cancer, cardiovascular disease, underlying medical condition, liver disease, diabetes, immunocompromised, neurological disorder, obesity, “other”, pregnancy, renal disease and tuberculosis. High-risk status was defined as individuals aged 60 years and older, immunocompromised, having cardiovascular conditions or COPD. Clinical symptoms were classified as asymptomatic, symptomatic or missing. Clinical outcomes were classified as ever hospitalized, ever in the intensive care unit (ICU) or death. Clinical outcomes were listed in hierarchical order (i.e. each case is counted with the highest-level outcome only, specifically: death, ICU, then hospitalizations).

We included all cases that were linked to a farm outbreak as a “farm worker” and this may include farm owners, family members, employees on the farm and individuals who visited the farm if they were deemed to be related to the farm outbreak based on the PHU investigation.

We used chi-square tests of proportions to compare medical risk factors outcomes of farm outbreak cases to overall laboratory-confirmed COVID-19 cases in Ontario aged 20–59 years (corresponding to approximately 95.0% of the cohort population) excluding farm-outbreak associated cases and long-term care home resident cases dated January 1 to December 31, 2020. Long-term care home cases were excluded given their differential risk and the nature of public health measures applied.

### Epidemiologic analysis

Descriptive statistics were used to describe COVID-19 farm outbreaks in Ontario. Proportions were calculated for categories of outbreak-associated cases by gender, age, medical risk factors, clinical presentation, outcomes and PHU. Outbreaks and outbreak-associated cases were further subdivided by season and the mean, median and range of the number, duration, and size of outbreaks was calculated for each season. Finally, the percentage of total farm outbreaks and outbreak-associated cases in greenhouses was calculated for each season. An epidemiologic curve was used to display outbreaks among the three PHUs with the most outbreaks, along with the number of outbreak-associated cases over the period included. Descriptive statistics were also used to describe non-farm outbreak associated cases. All analyses were conducted using SAS Enterprise Guide (version 9.4) and Microsoft Excel.

## Results

There were a total of 64 farm outbreaks with 2,202 outbreak-linked cases (**Table 1**). Outbreaks ranged in size from one to 240 cases (median 15 cases), with 63 outbreaks (98.4%) having two or more cases and six outbreaks (9.4%) having 100 or more cases. Outbreak duration ranged from zero days (i.e. all cases as part of the outbreak had the same episode date) to 128 days (median 23 days).

**Table 1:** COVID-19 farm outbreaks in Ontario, January 1–December 31, 2020

Overall outbreak descriptions	Frequency	Mean	Median	Range
Total number of outbreaks	64	N/A	N/A	N/A
Total number of outbreak-associated cases	2,202	34.4	14.5	1–240
Duration of all outbreaks (days)	N/A	31.3	23	0–128

Abbreviations: COVID-19, coronavirus disease 2019; N/A, not applicable

**Figure 1:**
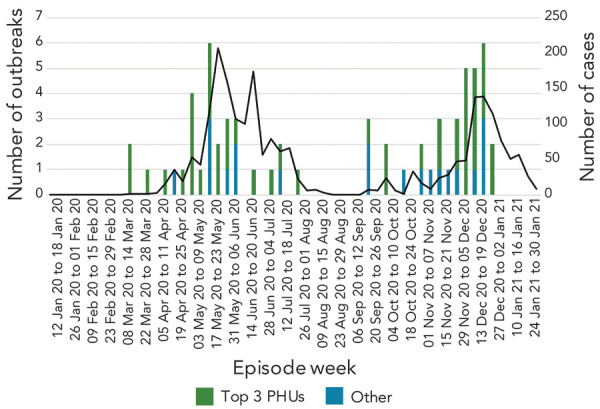
Epidemiologic curve of COVID-19 farm outbreaks in Ontario, January 1–December 31, 2020^a^ Abbreviations: COVID-19, coronavirus disease 2019; PHU, public health unit ^a^ Bars correspond to the number of outbreaks reported by the top three PHUs with the most COVID-19 on-farm outbreaks reported in Ontario and the total number of COVID-19 on-farm outbreaks reported by all other PHUs combined. The black line corresponds to total number of COVID-19 on-farm cases reported across all PHUs in Ontario. Top 3 PHUs correspond to PHUs with most outbreaks reported in the province: Windsor-Essex county Health Unit (N=35), Haldimand-Norfolk Health Unit (N=6) and Chatham-Kent Public Health (N=4)

A total of 37 (57.8%) farm outbreaks occurred on farms classified as greenhouses. The majority of farm outbreaks occurred in three PHUs (Windsor-Essex County Health Unit, Haldimand-Norfolk Health Unit and Chatham-Kent Public Health) that accounted for 68.8% (n=44/64) of all farm outbreaks in Ontario. Farm outbreaks peaked in May 2020 and again in December 2020. Farm outbreaks were infrequent from the end of June to early September 2020 (**Figure 1**).

When comparing COVID-19 farm outbreaks by season (**Table 2**), the total number of outbreaks was highest in the spring and fall (25 outbreaks each); however, the total number of outbreak-associated cases was highest in the spring (n=1,292 cases, 58.7%), followed by fall (n=772 cases, 35.1%) and summer (n=69 cases, 3.1%). Duration of outbreaks was also longest for outbreaks starting in the spring, with a mean duration of 43.2 days (range: 1–128 days), followed by fall (mean duration 29.1 days [range: 0−76 days]) and summer 14.2 days (range: 4–29 days). There was a higher proportion of farm outbreaks occurring on farms classified as “greenhouses” in the summer (83.3%) and spring (68.0%), compared to fall (52.0%). The majority of outbreaks occurred in Windsor-Essex, where there is a high density of agricultural farms, regardless of the season.

**Table 2:** COVID-19 farm outbreaks in Ontario by season^a^, January 1–December 31, 2020

Description of outbreaks and outbreak-associated cases		Spring (March 20–June 19)	Summer (June 20–Sept 21)	Fall (Sept 20–22–Dec 20)	Winter (Dec 20–31)^b^	Total
Total outbreaks		25	6	25	8	64
Total outbreak-associated cases	Total (N)	1,292	69	772	69	2,202
	Percent of total (%)	58.7%	3.1%	35.1%	3.1%	100%
	Mean cases per outbreak (N)	51.7	11.5	30.9	8.6	34.4
	Median cases per outbreak (N)	21	6.5	25	4	14.5
	Range of cases per outbreak (N)	2–240	3–30	3–77	1–27	1–240
Duration of all outbreaks (days)	Mean	43.2	14.2	29.1	13.8	31.3
	Median	38	14.5	24	11	23
	Range^c^	1–128	4–29	0–76	0–34	0–128
Greenhouses	Total outbreaks (N)	17	5	13	2	37
	Percent of total outbreaks (%) by season	68.0%	83.3%	52.0%	25.0%	57.8%
	Outbreak-associated cases (N)	822	63	409	46	1,340
	Percent of total cases (%) by season	63.6%	91.3%	53.0%	66.7%	60.9%

Abbreviation: COVID-19, coronavirus disease 2019

^a^ There were no farm outbreaks between January 1 and March 19, 2020

^b^ Data for winter is limited due to the study period ending December 31, 2020; data is included here for overall comparison, but not included in the analysis and discussion

^c^ Zero days indicates that all cases as part of the outbreak had the same episode date

Outbreak-linked cases were predominantly male (83.2%) with a median age of 35 years. There were 221 (10.0%) cases that had one or more comorbidities and 121 (5.5%) that met criteria for high-risk status. The majority were symptomatic (n=1,375; 62.4%), while 688 (31.2%) were asymptomatic and symptoms were missing in 139 (6.3%) cases. In total, there were 16 (0.7%) outbreak-associated cases that were hospitalized, eight (0.4%) cases admitted to the ICU and three (0.1%) deaths. The majority of outbreak-associated cases were associated with three PHUs with 1,498 (68.0%) from Windsor-Essex, 260 (11.8%) from Haldimand-Norfolk and 143 (6.5%) from Chatham-Kent (**Table 3**).

**Table 3:** Characteristics of farm outbreak-associated cases for outbreaks dated January 1–December 31, 2020

Outbreak-associated cases	Frequency	Proportion
Total	2,202	N/A
**Gender**			
Male	1,831	83.2
Female	332	15.1
Unknown or missing	39	1.8
**Age (years)**			
Younger than 10	1	0.0
10–19	23	1.0
20–29	672	30.5
30–39	740	33.6
40–49	467	21.2
50–59	204	9.3
60–69	81	3.7
70–79	11	0.5
80 and older	1	0.0
Unknown	2	0.1
**Medical risk factors**			
One or more comorbidities^a^	221	10.0
High-risk status^b^	121	5.5
**Clinical presentation**			
Asymptomatic	688	31.2
Symptomatic	1,375	62.4
Missing symptoms	139	6.3
**Outcomes^c^**			
Death	3	0.1
ICU	8	0.4
Hospitalized	16	0.7
**Public health unit where outbreak occurred^d^**			
Chatham-Kent Public Health	143	6.5
Haldimand-Norfolk Health Unit	260	11.8
Halton Region Public Health	82	3.7
Middlesex-London Health Unit	31	1.4
Niagara Region Public Health	83	3.8
Region of Waterloo Public Health and Emergency Services	18	0.8
Simcoe Muskoka District Health Unit	24	1.1
Southwestern Public Health	44	2.0
Windsor-Essex County Health Unit	1,498	68.0

Abbreviations: COPD, chronic obstructive pulmonary disease; ICU, intensive care unit; N/A, not applicable

^a^ Includes: anemia, asthma, COPD, cancer, cardiovascular disease, underlying medical condition, liver disease, diabetes, immunocompromised, neurological disorder, obesity, “other”, pregnant, renal disease, tuberculosis

^b^ Includes: Age 60 years and older, immunocompromised, cardiovascular condition, COPD

^c^ Listed in hierarchical order (i.e. each case is counted with the highest-level outcome only)

^d^ Three public health units with fewer than 15 outbreak-associated cases were not presented in the table

Compared with farm outbreak-associated cases, cases in the general population (n=177,092) had more comorbidities (n=29,620, 16.7%, *p*<0.05) and a higher proportion were hospitalized (n=2,733, 1.5%, *p*<0.05). The proportions of cases that were admitted to the ICU (n=651, 0.4%, *p*=0.49) or died (n=237, 0.1%, *p*=0.45) were similar.

## Discussion

Farm outbreaks of COVID-19 in Ontario occurred throughout most of 2020 with increased activity in the spring and fall, and were associated with 2,202 cases, 16 hospitalizations and three deaths. Farm outbreaks peaked in May 2020 and December 2020 corresponding to the increased rates of COVID-19 cases in the province overall ((11)). The spring peak occurred after the March 2020 implementation of travel restrictions and stay-at-home orders ((12)), and prior to the implementation of provincial farm outbreak guidance and other public health measures in Ontario issued in September 2020. During the summer months, when COVID-19 transmission was low in the province, there were fewer outbreaks overall and the majority of outbreaks occurred in greenhouses. The indoor and crowded nature of greenhouse work, at a time when indoor masking was not routinely recommended or used, may have promoted transmission of COVID-19 and could have contributed to outbreaks on farms even when there was lower levels of community transmission. The relative role of indoor, crowded daytime working conditions of greenhouses compared to other risks of transmission on farms, such as congregate living among workers, warrants further investigation.

In a previous study of workplace outbreaks and outbreak-associated cases in Ontario, the agricultural sector had among the highest incidence rates of COVID-19 per hours worked compared to other labour force sectors ((13)). Additionally, for the time period of April 1 to August 31, 2020, the agricultural sector had the second-highest proportion of outbreak-associated cases and cases that were hospitalized compared to other industry sectors ((13)). This analysis specifically focuses in on characteristics of farm-related outbreaks and cases to describe their unique characteristics and explores factors that may have contributed to the over-representation of the agricultural sector for outbreaks, specifically greenhouse farms, and their potential role in contributing to farm outbreaks. A previous study of workplace outbreaks in Ontario has also shown that individuals associated with workplace outbreaks are younger, healthier and have lower rates of severe outcomes compared to the general population ((14)). In comparison to previously published workplace (all industries) outbreak-associated cases, farm outbreak-associated cases were younger, had fewer comorbidities, and had a lower proportion of hospitalizations and deaths. However, in this analysis, compared to the general population of cases of the same age, farm outbreak cases had similar proportions of ICU and death outcomes, despite a lower proportion with comorbidities. This suggests that there were differential risks for the most severe outcomes for farm outbreaks compared with other workplace outbreaks.

A number of previous studies cite challenges for farm workers which may contribute to higher rates of COVID-19 outbreaks on farms. In New York state, it was noted that farm workers did not have adequate access to personal protective equipment until COVID-19 infections were at an “alarmingly high rate” ((15)). Fear of job loss and deportation, lack of income replacement programs while isolating or sick, language and cultural barriers and having long and irregular hours are believed to contribute to farm workers avoiding testing or treatment ((5,16)). This is of particular importance in Ontario, as 31% of farm workers are also temporary foreign workers, with limited access to resources ((3)). It has also been noted that there are deficiencies in housing standards in many jurisdictions in Ontario, including windows that cannot open (limiting ventilation), inadequate laundry facilities (for cleaning work clothing) and high occupancy (limiting physical distancing in sleeping quarters and other shared facilities) which may contribute to spreading of COVID-19 among farmworkers ((17)).

## Limitations

The epidemiologic analysis of this study is subject to limitations. Firstly, only data entered into CCM were available for analysis. The number of cases of COVID-19 in CCM was subject to varying degrees of underreporting as not all individuals with COVID-19 developed symptoms, sought medical attention or testing and, therefore, the disease may have been unreported. Therefore, the number of outbreak-associated cases for each outbreak was likely an underestimate. As well, four outbreaks were classified as “open” as of data extraction and the data for these outbreaks is potentially subject to change. Misclassification of greenhouse status is possible as it was manually coded. Additionally, data in CCM does not specify where on the farm the outbreak occurred and cases may be unrelated to the greenhouse setting. This can make it difficult to draw definite conclusions about greenhouse farms. Other potential factors associated with outbreaks, such as local quarantine procedures, number of foreign workers, number of people living in shared housing, were not available for analysis.

## Conclusion

With the introduction of COVID-19 vaccines and workplace infection prevention and control measures over the course of the pandemic, the risk of large and long farm outbreaks has significantly reduced. However, given the relaxation of public health measures, including indoor masking, the return of international travel and the ongoing risk of the emergence of a new and more transmissible variant of concern, farms may continue to be settings vulnerable to COVID-19 outbreaks. Future studies are needed to understand the role of greenhouse work and other factors that may contribute to farm outbreaks of COVID-19.
